# Liquid Biopsy in Clinical Management of Breast, Lung, and Colorectal Cancer

**DOI:** 10.3389/fmed.2018.00009

**Published:** 2018-01-30

**Authors:** Ivana Bratić Hench, Jürgen Hench, Markus Tolnay

**Affiliations:** ^1^Institute for Medical Genetics and Pathology, University Hospital Basel, Basel, Switzerland

**Keywords:** liquid biopsy, cell-free DNA, ctDNA, circulating tumor cell, cancer, screening, blood

## Abstract

Examination of tumor molecular characteristics by liquid biopsy is likely to greatly influence personalized cancer patient management. Analysis of circulating tumor DNA (ctDNA), circulating tumor cells (CTCs), and tumor-derived exosomes, all collectively referred to as “liquid biopsies,” are not only a modality to monitor treatment efficacy, disease progression, and emerging therapy resistance mechanisms, but they also assess tumor heterogeneity and evolution in real time. We review the literature concerning the examination of ctDNA and CTC in a diagnostic setting, evaluating their prognostic, predictive, and monitoring capabilities. We discuss the advantages and limitations of various leading ctDNA/CTC analysis technologies. Finally, guided by the results of clinical trials, we discuss the readiness of cell-free DNA and CTC as routine biomarkers in the context of various common types of neoplastic disease. At this moment, one cannot conclude whether or not liquid biopsy will become a mainstay in oncology practice.

## Introduction

Testing of bodily fluids in medical diagnostics has a long history with Greek humorism and the Indian Ayurveda system as prominent examples. These antique ideas remain applicable since analysis of bodily fluids can reveal diseases. “Liquid biopsies” are commonly blood and urine samples and the term usually refers to neoplasia diagnostics in analogy to classical biopsies. The most prominent example of a “liquid biopsy” is testing for prostate-specific antigen (PSA) in blood samples, which, if obtained *lege artis*, is a robust predictor of prostate cancer. However, its specificity can be hampered by non-neoplastic prostate damage since PSA is present in both cancer and normal cells. Many cells and tissues release some of their constituents to the bloodstream, including fragmented, cell-free DNA (cfDNA) which can also arise from tumor cells, i.e., circulating tumor DNA (ctDNA). As opposed to PSA and other proteins, ctDNA sequences are tumor specific. Such changes have been associated with a variety of neoplastic, but also as hereditary disorders. The BRAF(p.V600E) point mutation, e.g., is found in a wide range of malignant and benign neoplastic diseases in many organ systems. Other mutations, such as MYD88(p.L265P), have a narrower occurrence spectrum restricted to hematological disorders. Lastly, some mutations, such as in the VHL gene, occur both in sporadic but also hereditary hemangioblastomas.

In contrast to PSA and alike, the diagnostic value of ctDNA dramatically increases with prior knowledge about the mutational landscape of a tumor based on a conventional biopsy. Targeted NGS reveals traceable mutations. With these patient-specific data at hand, liquid biopsies, designed to determine the exact amount of ctDNA fragments in blood, may become an invaluable personalized surveillance tool. The ultimate goal of precision medicine is to deliver the most suitable, personalized cancer treatment at the most appropriate dose and time point to maximize quality of life and OS. Liquid biopsies might pave the road toward this aim, offering a way to quantify treatment response and detect emerging resistance in real time without the need of serial conventional biopsies.

Herein, we will discuss the use of cfDNA/ctDNA as diagnostic, prognostic, and predictive biomarkers and their applicability in clinical settings. In addition, the modalities by which circulating tumor cells (CTCs) can be detected in peripheral blood and to which extend this principle has been adopted in clinical practice will be reviewed. Due to the overwhelming number of studies, we will, for the sake of data comparability, focus on the three most frequently investigated diseases: breast, lung, and colorectal cancer.

## cfDNA and ctDNA

### Biology and Origin of cfDNA/ctDNA

Cell-free DNA gained increased attention upon the discovery that part of it originates from tumor cells and can be isolated from peripheral blood (here: ctDNA), urine, and other bodily fluids (1). The amount of ctDNA likely depends on tumor burden. It was estimated in CRC that a tumor load of 100 g, corresponding to 3 × 10^10^ neoplastic cells, would release 3.3% of tumor (tissue) DNA into the blood daily (2). Mutations found in cfDNA are likely to represent a mixture of alterations in primary tumor and/or metastatic sites (3). The discovery that blood-derived cfDNA contains tumor-specific genetic and epigenetic alterations has provided a solid ground to clinical usage of ctDNA as a biomarker. Most of the respective studies showed high concordance between individual mutations found in tDNA and cfDNA samples (4). There is evidence that ctDNA analysis could inform about clonal heterogeneity and subclonal changes in real time (5–8). In healthy subjects, 70–90% of the cfDNA pool originates from white blood cells (mostly neutrophils and lymphocytes). Neoplastic cells, tumor-infiltrating T-lymphocytes, and degenerating endothelial cells found in the vicinity of expanding carcinoma tissue, likely contribute to cfDNA (9). However, the exact mechanisms of how cfDNA is released remains elusive. Three major hypotheses of ctDNA origin exist: (i) from dying tumor cells; (ii) from CTCs, and (iii) *via* active release. The majority of ctDNA fragments is likely derived from disintegrating cells (i.e., apoptosis, oncosis, and necrosis). Apoptosis is suggested to generate DNA fragments of about 180 bp and multiples of this length, appearing as ladder pattern in electrophoresis. Necrosis should result in longer fragments (>10.000 bp). Apoptosis, however, is likely impaired in most neoplasms. NGS to characterize plasma cfDNA profiles at single base resolution revealed that most cfDNA fragments in hepatocellular carcinoma (HCC) patients, healthy subjects as well as individuals with hepatitis B virus infections with and without cirrhosis, had a peak size near 166 bp. This length corresponds to DNA wrapped around a nucleosome and, thus, may be due to caspase-dependent endonucleases (10), supporting the idea that apoptosis is indeed a major source of cfDNA release. Atomic force microscopy of plasma cfDNA in stage IV CRC and healthy controls (11) indicated that over 80% of cfDNA fragments are shorter than 145 bp in CRC with no cfDNA fragment being larger than 300 bp. Likewise, controls had 65% fragments <145 bp but also 10% >300 bp. Blood-borne nuclease activities are likely responsible for this cfDNA size range. The discrepancies strongly argue against the diagnostic use of fragment length patterns. Many studies found larger sized DNA fragments and increased amounts of cfDNA in the plasma during advanced cancer stages and cytotoxic treatment, suggesting necrosis as release mechanism (2, 12–15). It was found that ctDNA fraction within cfDNA are enriched in short fragments (100–300 bp) while the same tumor-specific ctDNA mutations were not present in longer (1,000 bp) fragments (2, 15). Since most studies focused on frequent tumor types and were entity specific, the major contributor of ctDNA release remains elusive, might depend on cancer type, and could even also vary between patients (16). Further discrepancies among studies stem from different preanalytical conditions (serum vs. plasma, cfDNA extraction protocols), patient selection (varying tumor load and cancer stages) and the diversity of analytical methods. cfDNA fragments are likely protected from nuclease cleavage due to their association with nucleosomes (16). Determination of the nucleosome occupancy profile in cfDNA could potentially determine its tissue origin which might be beneficial in localizing “cancers of unknown primary” (17). ctDNA could originate from CTCs too. However, ctDNA levels are typically too high considering the low CTC counts in blood samples, and ctDNA is also present in absence of CTCs, making this hypothesis rather doubtable (4). Lastly, spontaneous, active release of ctDNA by tumors is the least investigated possibility. Such released ctDNA might have the role of an intercellular messenger and could either integrate into the genome of a host cell leading to genetic instability or it would bind to receptors leading to transformation of target recipient cells at distant locations. This effect gave rise to the theory of “genometastasis” (18–21). An *in vitro* study on breast cancer cell lines showed that active cfDNA release, at least partially, occurs *via* exosomes which would further regulate proliferation of the neoplastic cells (22). Nevertheless, further work is needed to identify the underlying mechanisms and, more importantly, the biological significance of active cfDNA release.

While cancer patients generally have much higher cfDNA levels than healthy subjects, the total amount varies considerably even among patients with comparable cancer type and stage (23, 24). Nevertheless, direct correlations between ctDNA level and tumor burden, stage, vascularity, cellular turnover, and therapy response were reported for various neoplasms (4, 15, 23, 25). Opposedly, increased cfDNA levels are not specific to cancer, as they are equally found in various pathologic (e.g., chronic inflammation, autoimmune disease) (26) and physiologic conditions (e.g., extensive exercise) (27), making it difficult to use cfDNA levels as a cancer-specific biomarker.

Knowledge about cfDNA clearance mechanisms comes from prenatal diagnostics. Clearance of fetal DNA from maternal blood occurs in two phases: a first rapid phase with a mean half-life of ~1 h (28) and a second slow phase of ~13 h (29). It has been speculated that the liver, spleen, and kidney might be responsible for cfDNA elimination (30). Since ctDNA is much less defined than circulating free fetal DNA, further investigation is required to understand its removal from the blood.

### Methodology in cfDNA/ctDNA Analysis

#### Preanalytics

Comparison of cfDNA concentrations between different malignancies and estimation of the prognostic potential of cfDNA levels is currently impossible due to disparities among sample preparation techniques (plasma vs. serum, containers used for blood withdraw, storage conditions), cfDNA isolation (amount of blood, centrifugal speed, cfDNA isolation kits), and DNA concentration measurement (colorimetric/fluorometric assays, real-time PCR, Picogreen or SYBR Green I dsDNA quantification assays, PCR assays targeting different genes, etc.) (31–34). Moreover, the lack of generally accepted units for cfDNA quantification largely impairs comparative retrospective, and even prospective studies. The result from the first large-scale quality control external quality assessment (EQA) scheme that investigated the impact of preanalytical conditions on cfDNA quality, quantity, and integrity showed that different extraction kits produce a wide range of cfDNA yields ranging from 2.87 to 224 pg/μl (35). Moreover, results from the first EQA scheme for isolation and analysis of ctDNA that involved 42 laboratories from 10 European countries reported high variability in multiple phases of cfDNA processing and in choice of genotyping technologies with regard to cfDNA analysis and overall error rate of 6.09% (36).

Increased cfDNA concentrations have been observed more frequently in serum (3- to 24-fold higher) than in plasma from both healthy control subjects and cancer patients (37–40). These higher values are likely the result of *in vitro* hemolysis during clotting (37, 39). Higher cfDNA levels have been observed in advanced tumor stages than in patients with non-metastatic disease (23, 40). However, the increase in serum, but not plasma, cfDNA concentration in advanced tumor stages strongly correlated with leukocyte counts (40).

The current preanalytical recommendations for cfDNA analysis are as follows: (1) blood processing within 3 h if an EDTA Monovette^®^ (Sarstedt, Germany) or similar instrument is used. Otherwise, vacuum containers (e.g., Vacutainer^®^, Becton-Dickinson, USA) with stabilizing reagents should be used; (2) plasma should be isolated by at least two sequential centrifugation steps; (3) plasma should be stored at −80°C and frequent freeze–thaw cycles should be avoided; and (4) use of dedicated cfDNA extraction kits (41).

#### Analytics

The possibility to identify oncogenic driver mutations offers advantage of ctDNA testing over solid biopsies and conventional biomarkers (e.g. serum PSA, CA15, CEA, and so on) the latter of which are not causally involved in tumorigenesis and, hence, not specific for neoplasia. The technical challenge in ctDNA detection stems from its low (e.g., 0.01%) fraction of cfDNA (2), demanding high sensitivity and specificity techniques, such as qPCR (ARMS, see below), dPCR [BEAMing; Droplet Digital™ PCR (ddPCR™)], and targeted parallel sequencing (next-generation sequencing, NGS). PCR-based methods, such as Cobas (FDA, CE-IVD) and Therascreen (CE-IVD) assays, based on the Scorpion Amplification-Refractory Mutation System (ARMS) reaction, are able to detect single base changes or small deletions by use of allele-specific primers. qPCR readout is cut-off based distinction of presence or absence of the mutation in question, detecting AFs down to 1% (42–45). Their sensitivity is only moderate compared to dPCR and targeted NGS with detection thresholds around 0.1% or lower (46, 47).

Droplet Digital™ PCR (ddPCR™, Bio-Rad or RainDrop™, RainDance Technologies) and BEAMing (beads, emulsion, amplification, magnetics; Sysmex) are two PCR-based techniques based on a similar principle, where DNA is diluted down to single DNA molecules that get physically separated into individual reaction compartments. Before quantification, DNA templates are amplified separately either on beads (BEAMing) or in water droplets engulfed by oil (ddPCR). Readout—analogous to flow cytometry—detects AFs down to 0.005% (4, 48, 49). Despite a sensitivity of BEAMing comparable to ddPCR™, the more complex protocol limits its clinical use. While the concentration of mutant fragments detected by qPCR is calculated relatively to a standard curve, ddPCR™ allows an end point analysis in a cell counter-like manner. The advantage of digital approaches is their high specificity, high sensitivity, speed, independence of qPCR equipment, ease of use, and combined with comparatively low cost. A specialized droplet/bead reader is required for ddPCR™ but not for BEAMing which works on flow cytometers. The major disadvantage of qPCR and dPCR is their restriction to predefined genetic alterations unlike NGS that is able to detect novel changes without modification of the protocol. Yet, given current clinical consequences, detection of previously unreported alterations is neither required nor helpful for therapeutic decisions. To monitor the disease after determining distinctive mutations, specific probes can be individually designed as a personalized assay for peripheral blood samples, potentially beneficial in early recurrence detection, disease progression monitoring, and identification of resistance mechanisms prior to conventional clinical signs (43, 49, 50).

NGS detects multiple somatic mutations in plasma cfDNA and informs about intratumoral heterogeneity, potentially useful in metastatic disease. However, the sequencing error rate of conventional NGS approaches is too high for detection of rare cfDNA variants. The Illumina^®^ platform has error rates from 0.29 to 1% depending on read length, library preparation, base misincorporation, base calling algorithms, and variant type (6, 51, 52). Therefore, adaptations were made for ultrasensitive detection of low mutation AFs, in particular, an alternative library preparation, read depth, and coverage. Targeted gene panels infer high coverage per base at moderate cost. CAncer Personalized Profiling by deep Sequencing (CAPP-Seq) is a capture-based NGS ctDNA detection method for SNVs, indels, rearrangements, and CNVs (47). This method enriches for recurrently mutated genomic regions chosen for specific cancer types prior to sequencing by hybridization to a pool of antisense biotinylated oligonucleotides. High coverage (e.g., 10,000×) sequencing of the captured DNA detected ctDNA in 100% of stage II–IV and 50% of stage I non-small cell lung cancer (NSCLC) patients, with 96% specificity for mutant AFs down to 0.02% (47). Even though no patient-specific customization is needed, the multi-phase bioinformatics framework for CAPP-Seq data analysis is rather unattractive for clinical use. Recently, the detection limit of CAPP-Seq was decreased to 0.004% AF by incorporating molecular barcoding and iDES (53). The alternative Safe-Sequencing System (Safe-SeqS) detects variants down to 9/10^6^ by assignment of UIDs to each DNA molecule prior to PCR amplification. The number of molecules in the sample is estimated based on UIDs and precisely quantified. A variant is called only if contained in >95% of PCR fragments within the same UID family (52). Polymerase and oligonucleotide synthesis errors can, hence, be excluded, making it possible to detect a mutant AF of 0.0001%. Safe-SeqS furthermore corrects for the PCR amplification bias. Its disadvantages are the relatively long turnaround time and the possibility of false-positives due to imperfect amplification (52). targeted error correction sequencing (TEC-Seq) is an alternative approach based on targeted capture of multiple regions of the genome labeled with dual-index barcode adapters and subsequent deep sequencing (30,000×) with an analytical specificity of >99.9999% and sensitivity of 97.4%. Lower false-positive rates (< 3 × 10^−7^) comparable to iDES were reported (54). Evaluation of 200 plasma samples with TEC-Seq detected somatic mutations in 71, 59, 59, and 68% of patients with early-stage breast, colorectal, lung, and ovarian cancer (54). Importantly, the detection limit depends on the number of free DNA molecules present in a sample. A typical plasma sample of 1 ml contains approximately 3,000 copies of any given gene leading to a sensitivity limit for detection of only 1 in 15,000 copies in 5 ml (55). Despite the low detection limits observed in CAPP-Seq, iDES, Safe-SaqS, and TEC-Seq they have, to our knowledge, only been systematically tested in research settings. Besides the Illumina platform, semiconductor-based targeted Ion Torrent™ sequencing (Thermo Fisher) has been successfully applied in analysis of cfDNA, too (56–58).

Only few laboratories have analyzed genome-wide copy number alterations, rearrangements, and mutations in cfDNA. Shotgun massively parallel sequencing method was applied to detect CNVs and point mutations in plasma of a four-patient case study with HCC and two patients with synchronous breast and ovarian cancers. Changes in ctDNA level in pre- and post-surgery blood samples were tracked to monitor disease burden during tumor evolution (59). The largest tumor had the highest ctDNA level and most CNVs (59). Plasma ctDNA WGS performed on 10 CRC and BC patients (60) demonstrated the feasibility of detecting chromosomal aberrations. WES of cfDNA in advanced cancers was able to objectify tumor evolution in response to therapy. Plasma cfDNA collected at the beginning of treatment and at relapse in 4/6 patients was analyzed (3). Since the mutations were present at high AFs due to metastatic state, the study’s clinical significance is limited. WES of six NSCLC stage III patients identified a median of 17.19% of tumor variants in serum (61). Interestingly, a median of 1,218 additional variants were present in serum only and of unknown origin. The average 68.5× WES sequencing depth would be unable to detect commonly low ctDNA AFs. The key advantage of WGS and WES is their general applicability without personalization.

Only small amounts (200 μl–2 ml) of plasma were used for cfDNA extraction in the abovementioned studies, making it unlikely to detect low AFs (51): (i) typically low ctDNA counts prohibit high throughput approaches and could lead to artifacts, in particular false-positives. (ii) The fraction of false-negatives correlates with the sensitivity of the method. (iii) In order to achieve high sensitivity and specificity, normal reference samples (skin, lymphocytes, etc.) need to be analyzed concomitantly. However, imperfect sequencing of normal tissue would induce false-positives (4). Besides determination of extensive genetic profiles of the tumors in individual patients, WGS and WES remain unable to identify the origin of ctDNA and currently remain confined to translational research. Here, they offer the potential of discovering novel pathogenic variants to be tested in clinical trials. Their long turnaround time, need for extensive bioinformatic support, and lack of clinical associations for the majority of genomic alterations preclude their routine use.

DNA methylation signatures differ between tissues, allowing to determine their relative contributions to cfDNA by genome-wide bisulfite sequencing (8). Tissue of origin of genomic aberrations identified in cfDNA from 29 HCCs was elucidated by deconvolution of plasma bisulfite sequencing data into tissue contribution percentages. HCC patients had a higher liver contribution than healthy controls (8). This approach might, thus, be diagnostically helpful in identifying the origin of elevated cfDNA levels.

The multitude and diversity of techniques aimed at obtaining similar molecular information has led to discordant and even conflicting data. The majority of authors measured artificially spiked healthy donor blood samples to define sensitivity and specificity of their methods. Being a good starting point, it remains problematic that patient samples are hard to preserve in their native state which makes testing of multiple methods on the same specimen almost impossible. Furthermore, the ctDNA fraction of cfDNA is low, requiring relatively large blood volumes per test. SNP-based results from different platforms vary markedly (62). Physicians started to request confidence levels for clinical use of cancer genetic results and only fraction of them routinely requests genomic tumor profiling (63). Targeted approaches, currently featuring highest sensitivity and specificity, are likely the first to find acceptance among oncologists for disease monitoring.

### cfDNA/ctDNA in Cancer Patient Management

Circulating tumor DNA is present in over 75% of patients with advanced pancreatic, ovarian, colorectal, bladder, gastroesophageal, breast, melanoma, hepatocellular, and head, and neck cancers while rates below 50% were found in primary brain tumors, in renal, prostate, and thyroid cancers as well as in patients with localized tumors (4). Herein, we discuss the potential use of ctDNA as biomarker in a (i) diagnostic, (ii) prognostic, and (iii) predictive setting. The regimes comprise (a) total cfDNA level, (b) identification of tumor-specific genetic alterations, and (c) quantification of mutant alleles. Depending on cancer type, different combinations of these strategies might prove beneficial. The largest and—in our opinion—most relevant studies are summarized in Tables 1–3.

**Table 1:** Breast cancer.

Stage	Finding	Known Mut.	Method	Patient number	Reference
eBC	Circulating tumor DNA (ctDNA) can be detected in eBC before and after surgery	Yes	ddPCR	29	Beaver et al. (50)

I–III	ctDNA mutation level as prognostic factor for recurrence-free survival (RFS) and overall survival (OS)	Yes	ddPCR	110	Oshiro et al. (64)

eBC	Presence of PIK3CA mutations in cell-free DNA (cfDNA) prognostic of RFS and breast cancer-specific survival (BCSS) in TNBC	Yes	ddPCR	49	Takeshita et al. (65)

II–III	Presence of ctDNA in TNBC patients with residual disease correlates with inferior disease-free survival (DFS)	Yes	NGS	33	Chen Y-H et al. (66)

eBC	Postoperative ctDNA mutation level predictive of early recurrence	Yes	ddPCR NGS	55	Garcia-Murillas et al. (67)

n.s	Aberrant methylation of six genes (SFN, P16, hMLH1, HOXD13, PCDHGB7, and RASSF1a) in serum cfDNA of breast cancer patients in comparison to the healthy subjects and those with benign breast disease	No	MethyLight	267 cancer 236 benign disease 246 healthy	Shan et al. (68)

I–II	EGFR, PPM1E, and eight gene-specific CpG sites showed significantly hypermethylation in cancer patients plasma cfDNA and were significantly associated with BC	No	bisulfite NGS	86 cancer 67 healthy	Li et al. (69)

I–III	cfDNA analysis of SNPs and CNV in plasma can distinguish between patients with breast cancer and healthy controls	Yes	SNP 6.0 array	65 cancer 8 healthy	Shaw et al. (70)

IV	Detection of PIK3CA driver mutation in plasma of mBC	Yes	BEAMing	49 retrospective 50 prospective	Higgins et al. (71)

mBC	Detection of TP53 driver mutation in plasma of metastatic TNBC	Yes	NGS	40	Madic et al. (72)

III–IV	Presence of mutations in plasma cfDNA and their AF correlated with progression-free survival (PFS)	Yes	NGS	100	Liang et al. (73)

mBC	ctDNA level had a wider dynamic range and better correlation with changes in tumor burden than CA15-3 or circulating tumor cells (CTCs)	Yes	TAm-Seq dPCR WGS	30	Dawson et al. (74)

I–III	Detection of ctDNA precedes clinical detection of metastasis in 86% of patients with an average lead time of 11 months. Patients with undetectable ctDNA postoperatively had a long-term DSF. ctDNA quantity was predictive of poor survival	Yes	WGS ddPCR	20	Olsson et al. (75)

mBC	High-depth NGS of plasma ctDNA useful for *de novo* mutation identification and monitoring of somatic genetic alterations during targeted therapy	Yes	Targeted MPS	1	De Mattos-Arruda et al. (7)

IV	Serial analysis of ctDNA analysis for early detection of resistance mutations	Yes	NGS ddPCR	54	Guttery et al. (76)

IV	ctDNA analysis for detection of ESR1 resistance mutations	Yes	NGS ddPCR	8	Chu et al. (77)

pT1–pT4, pN0–pN3, pM0	Presence of CTCs before and after chemotherapy associated with poorer DFS (*p* < 0.0001), breast-cancer-specific survival (*p* < 0.008), and OS (*p* < 0.0002).	No	CellSearch	2,026 before,1,492 after therapy	Rack et al. (78)

mBC	CTC count before treatment is an independent predictor of PFS and OS in mBC	No	CellSearch	177	Cristofanilli et al. (79)

mBC	CTC count has prognostic value for mBC patients receiving first-line therapy. Changing the chemotherapy after one cycle of first-line therapy does not improve OS in mBC patients	No	CellSearch	595	Smerage et al. (80)

mBC	CTC positivity associated with reduced OS, but not with PFS regardless of the molecular subtype. No difference in CTC detection rate between different subtypes of primary tumor (HER2 positive vs. HER2 negative/hormone positive vs. TNBC)	No	CellSearch AdnaBreast	254	Müller et al. (81)

mBC	Presence of heterogeneity in PIK3CA mutational status between single CTCs in an individual patient	No	CellSearch DEPArray	39	Pestrin et al. (82)

mBC	10 single CTCs are sufficient for determination of HER2 status. In HER2-negative primaries, CTC can acquire HER2 gene amplification during disease progression	No	Immunomagnetic CTC enrichment Multicolor FISH Immunofluorescence	33	Meng et al. (83)

I–III	HER2-positive CTCs found in 89% (51/57) patients with HER2-negative primaries. Administration of trastuzumab can eliminate chemotherapy-resistant CK19 mRNA-positive CTCs, reduce risk of disease recurrence, and prolong the DFS	No	Cytospin Immunocytochemistry	75	Georgoulias et al. (84)

mBC	CNS-OR and 1-year OS was higher in patients with no CTC detected at day 21 before cycle 2 of systemic therapy	No	CellSearch	44	Pierga et al. (85)

**Table 2:** Lung cancer.

Stage	Finding	Known Mut.	Method	Patient Number	Reference
I–IV	Presence of plasma circulating tumor DNA (ctDNA) has higher positive predictive value than six biomarkers [CA125, CA19-9, CYFRA21-1, CEA, NSE, squamous cell carcinoma antigen (LSCC)]. Concordance rate between tDNA and ctDNA mutations was 78.1%. Decrease in AF of plasma ctDNA mutations observed 2 days postoperatively	Yes	NGS	41 non-small cell lung cancer (NSCLC) (33 LAC, 6 LSCC, 1 Neuroendocrine carcinoma, 1 LCC)	Guo et al. (86)

IA–B, IIA	Overall concordance rate between tDNA and cfDNA was 50.4%. cell-free DNA (cfDNA) level correlate with tumor stage. cfDNA has higher PPV for early-stage NSCLC than CA125, CA19-9, CEA, NSE, and CYFRA21-1 tumor markers	Yes	NGS	58 NSCLC (51 LAC, 7 LSCC)	Chen et al. (87)

I–IV	ctDNA detected in 100% of patients of stages II–IV, and in 50% of stage I	Yes	CAPP-Seq	17 NSCLC (14 LAC, 2 LSCC, 1 LCC)	Newman et al. (47)

IIIB, IV	78% of 97 of patients positive for EGFR variant in primary had these mutations in ctDNA; EGFR:p.L858R in either tumor tissue or cfDNA predicts shorter OS and progression-free survival (PFS). ctDNA level correlate with total tumor volume	Yes	TaqMan assay	97 NSCLC (91 LAC, 2 BAC, 1 LCC, 1 LSCC, 2 other)	Karachaliou et al. (88)

I–II	Methylation profiles of five genes (APC, CDH13, KLK10, DLEC1, and RASSF1A) in cfDNA of NSCLC patients showed a significantly higher tumor-specific hypermethylation frequency	Yes	Methylation-specific PCR	78 NSCLC (30 LAC, 36 LSCC, 12 other) 50 healthy	Zhang et al. (89)

I–IV	Plasma cfDNA level does not correlate with any particular histologic subtype of NSCLC, but with tumor stage. Significant correlation between plasma cfDNA concentration and lactate dehydrogenase (LDH) level. Patients with tumor progression have increase in plasma cfDNA concentrations but not in serum	No	Real-time PCR	185 NSCLC (81 LAC, 49 LSCC, 37 LCC, 18 undifferentiated) 46 healthy	Gautschi et al. (40)

I–III	Plasma cfDNA level does not correlate with sex, age, histotype, and tumor stage. Increased cfDNA level does not correlate with recurrence-free survival and overall survival (OS). Plasma cfDNA level can be used as a biomarker for possible relapse during follow-up	No	PCR	84 NSCLC (47 LAC, 25 LSCC, 12 other) 43 healthy	Sozzi et al. (90)

IIIB, IV	Total cfDNA level does not predict chemotherapy response. Higher cfDNA level at baseline associated with worse disease-free survival (DFS) and OS	No	Fluorometry	218 NSCLC (147 LAC, 43 LSCC, 28 LCC)	Tissot et al. (91)

II–IV	Therapeutically targetable driver and resistance mutations can be detected in ctDNA. Higher ctDNA concentrations highly associated with decreased OS	Yes	NGS	102 NSCLC (83 LAC, 4 LSCC, 12 poorly differentiated carcinoma, 3 other)	Thompson et al. (92)

IIIB, IV	Presence of ctDNA at diagnosis in 71% of patients; related to shorter OS. ctDNA clearance at first evaluation (6–8 week) after treatment initiation associated with objective response, longer PFS and OS	Yes	NGS ddPCR	109 NSCLC (98 non-LSCC, 11 LSCC)	Pécuchet et al. (93)

III, IV	cfDNA levels do not correlate with hypermetabolic tumor volume	No	qPCR PET-CT	53 NSCLC (33 LAC, 19 LSCC, 1 other)	Nygaard et al. (94)

I–IV	Plasma cfDNA concentration correlates with LDH activity and NSE level in small cell lung cancer (SCLC) and NSCLC	No	Labeling by nick translation	22 SCLC 46 NSCLC (19 LAC, 18 LSCC, 9 undifferentiated)	Fournié et al. (95)

IIIA–B, IV	Overall concordance rate of mutations between tDNA and cfDNA was 78.21%. SNV, indels and gene fusions (EML4-ALK, KIF5B-RET) can be detected in cfDNA by targeted sequencing	Yes	targeted sequencing	39 NSCLC (34 LAC, 5 LSCC)	Yao et al. (96)

II–IV	Patients with cfDNA positive for KRAS mutation have shorter PFS and OS and have lower response rate to the chemotherapy	Yes	Amplification-Refractory Mutation System (ARMS) qPCR KRAS DxS	246 NSCLC (150 LAC, 75 LSCC, 8 LCC, 13 other)	Nygaard et al. (97)

IIIB, IV	No significant differences between patients with KRAS mutation or wild-type KRAS status in serum cfDNA with regard to baseline patient characteristics, response rates, PFS, or OS	No	direct sequencing	67 NSCLC (29 LAC, 19 LSCC, 9 LCC, 10 undifferentiated)	Camps et al. (98)

IIB–IV	EGFR activating mutations detected in plasma cfDNA of 72.7% patients and EGFR T790M mutation in 43.5% of patients	Yes	BEAMing	44 NSCLC (43 LAC, 1 LSCC)	Taniguchi et al. (99)

IIIA, IIIB, IV	EGFR T790M mutation detectable in tumor biopsy (75%), cfDNA (80%) and circulating tumor cell (CTC, 70%) in patients progressing on EGFR-TKI therapy	Yes	hbCTC-Chip direct sequencing	42 NSCLC	Sundaresan et al. (100)

n.s	ORR and median PFS are similar in patients with T790M-positive plasma or T790M-positive tumor. Detection of resistance mutation in plasma is unlikely if the activating mutation is not detected	Yes	BEAMing	216 NSCLC	Oxnard et al. (101)

IIIB, IV	Qualitative and quantitative analysis of EGFR T790M mutation in plasma cfDNA can predict prognosis on EGFR-TKI therapy	Yes	DHPLC ARMS qPCR Digital array chip	135 NSCLC (130 LAC, 5 non-LAC)	Wang et al. (102)

I–III	Phylogenetic profiling of ctDNA useful to track emerging subclones responsible for resistance and relapse. Tumor volume correlate with AF of clonal variants	Yes	Multi-region exome sequencing Multiplex-PCR NGS	100 NSCLC (58 LAC, 31 LSCC, 2 Carcinosarc., 1 LSC, 3 adenosquamous carcinoma, 1 large cell neuroendocrine carcinoma) 24 NSCLC (16 LAC, 8 LSCC)	Abbosh et al. (103)

IIIA, IIIB, IV	CTC are detectable in stage IIIB and IV, but not stage IIIA of NSCLC	No	CellSearch	32 LSCC 31 LAC 5 poorly differentiated 33 other	Krebs et al. (104)

I–IV	Cytopathologic features of CTC are not different between various histologic subtypes of LC. CTC are detectable in 49% of NSCLC patients preoperatively	No	ISET	208 NSCLC (115 LAC, 54 LSCC, 19 LCC, 10 sarcomatoid carcinoma, 5 adenosquamous carcinoma) 39 healthy	Hofman et al. (105)

n.s	CTC count does not correlate with tumor volume. Activating EGFR and resistance EGFR T790M mutation could be detected in CTCs.CTCs count can be used for monitoring the tumor response to the therapy	Yes	EpCAM-functionalized CTC Chip	27 NSCLC (19 LAC, 8 LAC/BAC)	Maheswaran et al. (106)

n.s	CTC can be detected in the COPD patients and could be used as an early indicator of invasive LC	No	ISET	168 COPD	Ilie et al. (107)

IIIB, IV	CTC could be used as a source of tumor DNA for NGS detection of EGFR mutation. Genetic heterogeneity in CTCs	Yes	CellSearch NGS	37 NSCLC 10 BC 12 healthy	Marchetti et al. (108)

n.s	CNA-based classifier derived from CTCs analysis can distinguish chemorefractory and chemosensitive disease	No	CellSearch DEPArray NGS WGS	31 SCLC	Carter et al. (109)

**Table 3:** Colorectal cancer.

Stage	Finding	Known Mut.	Method	Patient Number	Reference
/	Cell-free DNA (cfDNA) level cannot be used as a biomarker to distinguish subjects with premalignant from those without endoscopic lesions. cfDNA concentration can predict adenocarcinomas in FOBT positive patients	Yes	Mutant-enriched PCR qRT-PCR	179 healthy, FOBT positive	Perrone et al. (110)

/	Mutations in K-ras gene detected in plasma cfDNA are associated with risk of colorectal cancer (CRC)	Yes	PCR	240	Kopreski et al. (111)

n.s	No correlation between CEA and plasma cfDNA level. No association between plasma cfDNA level and age and gender of patient, location and size of tumor, histologic grading and Dukes’ stage. Changes in cfDNA level can be used for monitoring recurrence and prospectively to identify high-risk patients	No	DNA Dipstick Kit	70 20 healthy	Frattini et al. (112)

B,C,D	CRC patients have higher cfDNA level than healthy subjects at the time of surgery. cfDNA level can be used to confirm the presence of recurrence or metastasis. Correlation of CEA and cfDNA level was observed	Yes	Mutant-enriched PCR Fluorescent-methylation specific PCR	70 20 healthy	Frattini et al. (113)

I–IV	Circulating tumor DNA (ctDNA) analysis reveals disease recurrence earlier than conventional follow-up (lead time 10 months). ctDNA superior over CEA in monitoring CRC	Yes	NGS ddPCR	11	Reinert et al. (114)

A, B, C, D	Combined analysis of cfDNA and CEA has higher diagnostic capacity in CRC than each of markers alone	No	qRT-PCR	75 75 healthy	Flamini et al. (115)

I–IV	Mutations in plasma cfDNA with AF >0.1% showed clinical utility in monitoring tumor burden in CRC. Median plasma cfDNA level of healthy individuals, endoscopically resectable tumors and advanced CRCs are 4.2, 6.8, and 9.2 ng/ml, respectively	Yes	NGS ddPCR	44 9 healthy	Sato et al. (116)

II–IV	Changes in ctDNA level used to follow tumor dynamics	Yes	BEAMing qRT-PCR	18	Diehl et al. (48)

I–IV	No correlation between ctDNA and CEA. High preoperative ctDNA level correlate with poor prognosis, shorter progression-free survival (PFS) and overall survival (OS)	Yes	TEC-Seq	42 44 healthy	Phallen et al. (54)

IV	Plasma cfDNA level correlate with plasma mutant KRAS level. No difference in cfDNA level between KRAS and wt-positive disease. Concordance rate of 78% for KRAS mutation between primary and cfDNA	Yes	ARMS qPCR	108	Spindler et al. (117)

IV	High concordance rate between plasma cfDNA and tumor for BRAF, KRAS, and PIK3CA	Yes	BEAMing qRT-PCR	503	Tabernero et al. (118)

IV	Presence of KRAS mutation in plasma, but not in tumor is strong prognostic factor for PFS and OS. Positive correlation between cfDNA and LDH, but not with CEA	Yes	TheraScreen KRAS mutation kit	140	Spindler et al. (119)

II	Detection of ctDNA after resection of colon cancer indicates residual disease and identifies patients at high-risk for recurrence. Serial ctDNA level is more sensitive in predicting radiologic recurrence than CEA levels	Yes	Safe-Seq	230	Tie et al. (120)

IV	KRAS mutation detectable in plasma cfDNA 10 months before radiographic progression	Yes	iPLEX assay Exome sequencing BEAMing direct sequencing	18	Misale et al. (121)

IV	Re-challenge with EGFR-specific antibodies causes increase and decrease in percentage of mutant KRAS clones	Yes	HMRA Sanger sequencing Pyrosequencing BEAMing ddPCR NGS qRT-PCR	100	Siravegna et al. (122)

IV	Longer PFS in patients who experienced more than 10-fold change in ctDNA level after cycle 1 of chemotherapy. No correlation between OS and fold change in cDNA was observed	Yes	MPS	53	Tie et al. (123)

IV	Metastatic colorectal cancer (mCRC) patients with liver metastases and poorer performance had higher CTC count. 26% of mCRC patients at baseline had unfavorable (>3 CTCs/7.5 ml blood) CTC count. CTC number as prognostic and predictive factor at baseline and during follow-up	No	CellSearch	430	Cohen et al. (124)

I–IV	CTCs have been detected in all stages of CRC. Increased number of biphenotypic and mesenchymal CTCs in later stages of CRC. Presence of CTC with mesenchymal phenotype correlates with disease severity	No	CanPatrol	1,203	Zhao et al. (125)

I–IV	No clinicopathologic variables are associated with CTC detection in non-metastatic patients. Detection of the CTCs in non-metastatic patients associated with shorter OS and PFS. CTC count associated with the stage of disease. Preoperative detection of CTCs is strong prediction factor for disease progression and survival	No	CellSearch	287	Bork et al. (126)

n.s	Intra- and interpatient heterogeneity of CTCs with regard to EGFR amplification/expression and mutation profile in BRAF, KRAS, and PIK3CA gene	No	CellSearch qRT-PCR	49 mCRC 32 non-mCRC	Gasch et al. (127)

I–IV	90% of patients had at least 1 CTC/3 ml blood and 7% of those had CTC clusters. CTC number correlates to the disease stage, but not to CEA and CA19.9 markers. droplet digital PCR (ddPCR) is preferable technique to analyze KRAS mutation status in isolated CTCs	Yes	ScreenCell MB ddPCR TaqMeltPCR High-resolution melting Sanger sequencing MPS	35	Denis et al. (128)

### Early Detection of Cancer in Clinically Healthy Subjects

The potential of ctDNA to detect presymptomatic early-stage cancer or even precursor lesions would be clinically advantageous but was infrequently studied in clinically healthy subjects. In a longitudinal study, TP53 and KRAS mutations in cfDNA of non-smokers and ex-smokers were correlated with occurrence of various neoplasms potentially caused by tobacco smoke and air pollution (129). Plasma cfDNA from 550 healthy subjects was tested for mutations in two genes (TP53/*n* = 550; KRAS/*n* = 1,098). Such events were detected at an average 20.8 (TP53) and 14.3 months (KRAS) before cancer diagnosis (129). Mutant detection did not correlate with total cfDNA amounts. cfDNA mutations in healthy subjects were good predictors of bladder but not lung, upper-digestive tract, and blood cancers (129). Since tumor tissue was not tested, it remains unknown whether the mutations were present in the tumor cells. Adversely, some of the TP53/KRAS mutations were also detected in some healthy subjects not developing cancer during follow-up (129). A mutation in codon 249 of TP53 [NM_000546.5 (TP53):c.747G>T (p.Arg249Ser)], common after dietary exposure to the high levels of aflatoxin B1, was detected in plasma of 4/8 patients at least 1 year before initial diagnosis of HCC (130). To determine the specificity of such *ab initio* approaches, blood from 134 healthy subjects was ultra-deep sequenced in 50 cancer-associated genes with an average 30,000× coverage and a detection limit for variant AFs of 0.001% (131). Somatic mutations in blood cells significantly contribute to the mutational load in cfDNA, suggesting to rather sequence both cfDNA and blood cells in parallel for background removal (131). This finding was independently replicated in 2,728 patients with various tumors (132). Somatic alterations accumulate in solid tissues and the hematopoietic system as a function of age (133). The Circulating Cell-free Genome Atlas (CCGA, Clinical Trial NCT02889978, sponsored by Illumina^®^) is a 5-year prospective, multicenter, observational study aimed at developing ctDNA blood tests for early cancer detection (134). Ultra-broad, ultra-deep NGS combined with machine learning may found a database on mutations in the blood of subjects with and without cancer (134). This unbiased strategy might later provide risk assessment based on blood screening.

#### Breast Cancer

Detection of oncogenic driver mutations in early-stage presurgical breast cancer might tremendously impact on clinical management. Oncogenic driver mutations were screened for in 29 patients with early-stage BC (I-III) positive for 1/3 PIK3CA mutations (p.H1047R, p.E545K, p.E542K) before and after surgery. Mutant AFs in blood before surgery were low (0.01–0.07%) with the exception of one case with 2.99% who relapsed 26 months later (50). The same cfDNA mutations were found in 22% of 110 stage I–III BC patients (64), indicating a prognostic value for ctDNA AFs: higher values correlated with shorter RFS and OS (64) which holds true for TNBC, too (65). ctDNA detection in stage II–III TNBC patients with residual disease after neoadjuvant chemotherapy predicts recurrence with high specificity, but moderate sensitivity (66), potentially due to low plasma volume (1 ml) and a non-optimized NGS approach. ctDNA content shows significant correlation with prognosis at early cancer stages, as opposed to conventional protein tumor markers (64). Postoperative plasma ctDNA abundance after neoadjuvant chemotherapy and surgery, but not baseline levels predicted early recurrence (67). 50% of relapsing patients were ctDNA-positive postsurgically which increased to 80% during follow-up. None of the relapse-free patients had a ctDNA-positive blood sample. Importantly, ctDNA detection had a median lead time of 7.9 months over clinical recurrence diagnosis (67).

Serum cfDNA was analyzed for promoter methylation of six genes (SFN, P16, hMLH1, HOXD13, PCDHGB7, and RASSF1a) by the qPCR-based MethyLight test in 749 patients with BC, benign breast lesions, and healthy subjects for early disease detection (68). The method is able to discriminate between cancer and health with 79.6% sensitivity and 72.4% specificity. Malignant and benign lesions were distinguished with 82.4% sensitivity and 78.1% specificity (68). Likewise, the methylation profiles of EGFR, PPM1E, and eight more CpG islands in plasma cfDNA were used as biomarker for early BC detection (69). Alternatively, genome-wide CNV screening in cfDNA could distinguish cancer from health during routine follow-up (70). Even though the aforementioned studies suggest cfDNA analysis as a screening tool in early-stage BC, larger prospective trials are required to determine its reliability.

#### Lung Cancer

Unfortunately, despite WHO-defined (135) LC entities, several recent studies on liquid biopsies use the old, outdated SCLC and NSCLC categorization and lack precise distinction between LAC and LSCC, which in terms of tumor biology and targeted treatment options would be more informative. We tried to comply with the WHO classification (135) wherever possible.

Lung cancer commonly presents at advanced stage due to the lack of screening. Therefore, many studies assessed cfDNA testing for early LC detection and for recurrence monitoring. Concordance rate between tumor DNA and ctDNA mutations in the pretreatment plasma at early-stage (I, II) NSCLC patients was 78.1% (positive predictive value 94.7%), making it an indicator of early-stage LC (86). 89.7% of 58 early-stage (I–II) NSCLC patients had increased cfDNA out of whom 60.3% were ctDNA-positive with tumor-specific mutations (87). Others detected ctDNA in 100% of stage II–IV and 50% of stage I NSCLC patients (47). Reasons for this discordance are differences in detection technologies, small tumor size and molecular analysis of potentially non-representative tumor sections (87). 78% of 97 advanced-stage (IIIB, IV) NSCLCs featuring an EGFR variant in the primary had the same mutations in ctDNA; EGFR(p.L858R) in either tumor tissue or cfDNA predicted shorter OS and PFS (88). Changes in ctDNA AFs were observed when comparing pre- and postoperative cfDNA: AFs drop 11.52% in stage Ia and 14.63% in stage Ib, but only 0.57% in stage IIa, and 0.13% in stage IIIa. This drop already occurs 2 days postoperatively (86). cfDNA may have a higher positive predictive value compared to serum protein tumor biomarkers for early-stage LC (86, 87).

Methylation profiling in early-stage NSCLC might become a diagnostic and prognostic biomarker in analogy to breast cancer: Aberrant methylation profiles of five genes (APC, CDH13, KLK10, DLEC1, RASSF1A) in cfDNA from 110 early-stage mixed entity NSCLC cases showed a significantly higher tumor-specific hypermethylation frequency when compared to healthy controls, reaching 83.64% sensitivity and 74% specificity to diagnose LC (89).

#### Colorectal Cancer

Approximately 50% of localized CRC patients will develop metastases (136). Although there has been dramatic decline in the number of cases due to screening, CRC incidence remains high. Therefore, cfDNA testing might further improve screening efficiency. Traditional serum protein biomarkers (e.g., CEA, CA19-9) lack high specificity and sensitivity. There is only a limited number of studies investigating cfDNA testing for early CRC detection. A study on 170 subjectively healthy patients positive for occult fecal blood assessed the predictive power of plasma cfDNA levels and ctDNA KRAS mutations (110). Adenocarcinoma, but not intraepithelial precursor lesions, including HGIN, could be detected based on these values alone. Yet, the KRAS mutant AF was low (3%) compared to the AF in the tumor itself (45% in AC and HGIN), leaving the positive predictive value of the test questionable (110). Prospectively collected plasma cfDNA of 232 patients subjected to colonoscopy was analyzed for KRAS mutations which had previously been identified in the tumor tissues of 35 patients. These mutations were detectable in cfDNA in 29 patients (81%). 39% of patients positive for a KRAS mutation in cfDNA had a KRAS mutant colorectal neoplasia (111), suggesting to add cfDNA testing for frequent CRC mutations in screening programmes despite restriction to well-known CRC genetic aberrations.

CEA is the only routine tumor marker for estimation of tumor burden and progression monitoring despite low sensitivity and specificity, being elevated in 40% of CRC cases only (137). Several studies found that cfDNA performed better than CEA (138). No correlation between plasma cfDNA and CEA level was found (112). 151 plasma samples from six relapsing and five non-relapsing CRC patients were analyzed by NGS and ddPCR. Detection of ctDNA in cfDNA may provide an average of 10 months lead time over detection of metastatic recurrence by CEA (114). Combining CEA and cfDNA testing further improved diagnostic power (115).

### cfDNA/ctDNA Level as a Prognostic Biomarker

Clinical use of cfDNA levels alone as cancer biomarker is currently not recommended due to its highly variable amount; a broad spectrum of cfDNA levels in healthy controls has been observed. In a large multicenter study on 776 healthy individuals, the mean plasma cfDNA concentration was 67 ng/ml with an exorbitant standard deviation of 405 ng/ml (139). Meta analysis of 39 studies revealed cfDNA concentrations as low as 2.5 to 27 ng/ml (24), prohibiting to make cancer diagnoses based on a cut-off value: Other phenomena including inflammatory processes or tissue decay unrelated to cancer are impossible to distinguish. If still considering cfDNA levels as diagnostic modality, blood withdraw and cfDNA concentration measurement would require stringent standardization (140). Therefore, most studies summarized below analyzed ctDNA rather than cfDNA concentrations alone.

#### Breast Cancer

Numerous studies assessed the prognostic value of cfDNA levels in BC (141). A concentration range from ~60 to 550 ng/ml plasma or serum was found, only partially overlapping with healthy controls in whom values ranged from 3 to 63 ng/ml (142, 143). Many studies agree that cfDNA levels increase in patients with malignant lesions and correlated with tumor size, lymph node metastasis, histopathological grade, and clinical stage (141, 144–147). Two laboratories could not confirm the association between baseline total cfDNA level, pathologic complete response to neoadjuvant chemotherapy, and OS (145, 148). These conflicting results could be due to the variety of preanalytical and analytical methods as well as differences in patient cohorts. While the prognostic usefulness of ctDNA levels at baseline in a cohort of 30 metastatic luminal type BC patients was documented, another cohort of 36 patients with metastatic TNBC failed to verify this (72, 74), suggesting cancer type and burden dependency. Several studies addressed the prognostic value of cfDNA integrity reflected by the ratio of longer to shorter fragments. Analysis of Alu DNA repeats by qPCR in the serum of 51 healthy women and 83 with stage I–IV BC before surgery showed that mean DNA integrity was higher in BC patients with stage II, III, and IV, but no significant difference between stage I patients and healthy controls could be observed (149). A second comparable study confirmed this (150) while a third showed the opposite with lowest cfDNA integrity in metastatic BC (151). In summary, cfDNA levels alone are currently unlikely to become a clinically useful prognostic biomarker for BC, mainly due to their overall inability to stratify malignancy, benign lesions, and health (142, 152).

Analysis of genetic alterations seems more promising in cfDNA research. PIK3CA mutations were screened for in cfDNA of mBC patients in retrospective (*n* = 49) and prospective (*n* = 60) cohorts and were detected in approximately 30% of subjects with 100% concordance between ctDNA and FFPE specimens (71). A high mutation concordance of 81% between ctDNA and FFPE tumor samples was observed in TNBC too (72). An increased PIK3CA mutant AF predicted a short PFS in a retrospective cohort of 100 advanced-stage (III–IV) BC cases (73).

#### Lung Cancer

A broad spectrum of cfDNA concentrations, ranging, from ~3.7 to 318 ng/ml plasma/serum in LC have been reported (142). cfDNA concentration in stage II NSCLC (51 LAC, 7 LSCC) was significantly higher than in stage I, where no association was found between cfDNA level and age, sex, smoking history, tumor histology, differentiation level, and vascular invasion (87). While some authors found the highest plasma cfDNA concentrations in stage IV and, thus, a correlation between cfDNA level and tumor stage (40, 95), other studies could not confirm this (90). cfDNA levels in LC patients are significantly lower during follow-up than before surgery, suggesting a way to objectify surgical effectiveness (23, 90). High plasma cfDNA concentrations at baseline were significantly associated with decreased OS and increased tumor progression rates (40, 91, 92). 105 NSCLC patients (98 non-LSCC, 11 SCC) were categorized according to ctDNA concentration at baseline into the tertiles “high” (>0.50 ng/ml), “intermediate” (0.027–0.50 ng/ml), and “low” (<0.027 ng/ml) (93). “High” ctDNA levels were associated with high tumor burden, liver metastases, and high proliferative indices. The median OS was 13, 13.4, and 21.5 months; the median PFS was 4.1, 5.7, and 10.4 months for the ‘high,” “intermediate,” and “low” groups (93). Meta analysis of 22 studies concerning stages III and IV treated with chemotherapy suggests that higher levels of cfDNA are significantly associated with a shorter PFS and OS (153). Other studies could not recapitulate this correlation in different tumor types (47 LAC, 25 LSCC, 12 other unspecified types) (90). It remains unclear whether total tumor volume correlates with ctDNA level (47), or not (94).

Several studies addressed the relation between cfDNA level and other prognostic follow-up markers. A highly significant correlation was observed between increased plasma cfDNA level, elevated LDH level, advanced tumor stage, and poor OS (40). The association between OS and cfDNA concentration, NSE levels, and LDH activity has been reported earlier for SCLCs and NSCLCs, respectively (95).

Taken together, studies on LC share the major conceptual shortcoming of mixing biologically and clinically distinct NSCLC entities which, combined with the diversity in preanalytical and analytical methods, make data rather inconsistent concerning cfDNA levels as a standalone prognostic marker and suggest rather limited benefits for clinical practice.

Many studies assessed qualitative cfDNA analysis to identify ctDNA. A large meta-analysis reviewing 25 studies (2,605 NSCLCs) found a high concordance rate in EGFR mutation profiles between blood and tumor tissue. The authors concluded that EGFR mutation positivity in blood could be used to guide treatment decisions for EGFR TKIs in advanced NSCLC (154). 39 advanced NSCLC patients (34 LAC, 5 LSCC, stages III–IV) had 78.21% overall concordance between tumor tissue and cfDNA (96); point mutations, indels, and gene rearrangements (EML4-ALK, KIF5B-RET) were considered. AFs of concordant point mutations and indels in EGFR, KRAS, and PIK3CA ranged from 0.3 to 52% in tumor tissue DNA and in plasma cfDNA from 0.2 11.6% (96). The feasibility to detect NSCLC-related driver mutations in EGFR, KRAS, BRAF, and PIK3CA in cfDNA was demonstrated (99, 155). Mutant EGFR ctDNA levels correlate well with clinical response stage III and IV LACs (156). There are conflicting data concerning ctDNA KRAS mutations: Some studies found KRAS mutant ctDNA to be associated with recurrence and shorter OS (97, 157) while others did not (98). Two large meta-studies suggest that the presence but not type of ctDNA KRAS mutations (variants unspecified) was associated with shorter OS in NSCLC (153, 158). In summary, qualitative assessment of plasma ctDNA seems more appealing for LC diagnostics than cfDNA levels, particularly in cases without molecular access to tumor tissue.

#### Colorectal Cancer

Colonoscopy remains the gold standard for early detection of CRC with sensitivity rates of >80% (159) which can be complemented by testing fecal occult blood for mutations, currently having a higher sensitivity than plasma cfDNA analysis. cfDNA levels are elevated in metastatic CRC and have prognostic value (138, 160). Median plasma cfDNA levels of healthy individuals, endoscopically resectable tumors, and advanced CRC differ (116); a broad spectrum has been reported (142). cfDNA quantification may be useful for monitoring early-stage CRC postoperatively (112). cfDNA levels progressively decreased in patients who became tumor-free after surgery, while it increased in those developing recurrence and metastasis (112). CRC patients with a postoperatively detectable ctDNA relapsed within 1 year whereas those negative for ctDNA had no recurrence (48). 31 CRC patients across stages I–IV were preoperatively tested for ctDNA mutation rates. Patients with mutant AFs higher than 2% had significantly shorter PFS and OS compared to those <2% mutant AF (54). KRAS mutant ctDNA increase correlates with decreased OS (4). Meta-analysis of 1,076 patients with mCRC treated with chemotherapy confirmed that baseline total cfDNA levels correlate with OS (160).

Circulating tumor DNA in CRC was qualitatively examined in numerous studies which found a high agreement of mutations between tumor tissue and ctDNA (117, 138). Data divergence stems not only from different methodologies used for mutation detection but also from different samples sizes, clinical settings, and intervals between tumor biopsies and blood withdraw. Analysis of 108 chemotherapy-refractory mCRCs demonstrated that cfDNA and KRAS mutant ctDNA levels at baseline show a clear correlation. Patients with plasma mutant KRAS levels above 75% had a disease control rate of 0%, whereas those with lower levels had a rate of 42% (*p* = 0.048), indicating that ctDNA KRAS mutation AF predicts disease behavior (117). The applicability of cfDNA as prognostic parameter and alternative modality for mutation detection before treatment in mCRC was demonstrated in the randomized CORRECT phase III trial (118): 166 mCRC patients received placebo and 337 were treated with the TKI regorafenib. Plasma cfDNA was collected before treatment and tumor DNA from archival tissue. Presence of KRAS, PIK3CA, and BRAF mutations was tested in cfDNA and tumor tissue with an overall concordance rate of 76% for KRAS, 88% for PIK3CA, and 97% for BRAF mutations. Mean mutant AF detected in plasma was 11.05% for KRAS and 8.23% for PIK3CA. Despite high interpatient variability, high plasma cfDNA concentrations were associated with shorter median OS and PFS in both placebo and regorafenib groups (118). Shorter PFS during regorafenib treatment in patients with ctDNA KRAS mutations compared to those without was independently reported (161). The prognostic value of KRAS mutations in ctDNA but not tumor tissue was repeatedly confirmed (117, 119).

The utility of postoperative ctDNA as an indicator of MRD was demonstrated in a prospective cohort of 230 patients with resected stage II CRC (120). In subjects without adjuvant chemotherapy, tumor-specific mutations in cfDNA were detected postoperatively in 7.9% (14/178) patients of whom 79% (11/14) relapsed after a median follow-up of 27 months. In 164 patients without ctDNA, only 9.8% (16/164) experienced recurrence (120). Postoperative ctDNA positivity correlated with reduced RFS: 0% for ctDNA-positives and 90% for ctDNA-negatives after 3 years. Postoperative ctDNA status had a higher impact on RFS than any individual clinicopathological risk factor or any combination thereof (120). Presence of ctDNA immediately after chemotherapy completion was associated with a high risk of radiological relapse (120). ctDNA positivity precedes radiological recurrence up to several months, leaving more time to adapt the next line of treatment. In addition, detection of tumor-specific mutations in cfDNA has a higher specificity than CT scans (120). A strong association between ctDNA-positivity, RFS, and OS in patients with CRC irrespective of tumor stage, study size, tumor markers, detection methods, and sample type (plasma vs. serum) was revealed in a meta-analysis of 9 studies (1,022 CRC patients) (162).

In summary, total cfDNA levels and detection of ctDNA feature a strong prognostic value in metastatic CRC and are directly related to disease burden. cfDNA level and presence of ctDNA are prognostic markers of RFS, PFS, and OS. Disease progression is reflected by increasing cfDNA and ctDNA levels. Rapid decline in cfDNA level and absence ctDNA in plasma after initiation of therapy reflect treatment response.

### cfDNA/ctDNA for Monitoring Treatment Response and Resistance Mechanisms

As discussed above, studies in colorectal, breast, and lung cancer have shown that ctDNA, rather than cfDNA, levels correlate with the clinical disease course and can precede clinical progression detected by imaging by weeks or months. Spatial and temporal genomic heterogeneity in primary tumors makes it nearly impossible to capture their complete genomic profile in a single biopsy. Analysis of tumor heterogeneity would require sequencing of multiple regions from the primary and metastases, which to date is difficult in clinical settings. ctDNA allows longitudinal evolution and heterogeneity tracking, relapse prediction, quantifying therapy response, and resistance identification as reviewed in the cancer type-specific sections below.

#### Breast Cancer

Matched tumor and serial plasma samples from a prospective cohort of 30 mBC patients receiving systemic therapy were tested for somatic genetic alterations in TP53 and PIK3CA; structural variants were identified by deep targeted amplicon sequencing and WGS (74). ctDNA levels in serial samples at intervals ≥3 weeks during follow-up were analyzed by personalized dPCR and tagged amplicon deep sequencing. ctDNA was detected in ≥1 samples in 29/30 patients while CA15-3 was positive in only 21/27 cases. Plasma levels of ctDNA mutations and structural variants but not total cfDNA showed dynamic patterns correlating with CT-morphological treatment responses. In 10/19 patients ctDNA increased in average 5 months before radiographic disease progression. In some cases, multiple ctDNA mutations exhibited similar AF dynamics, while in others, a ctDNA TP53 mutation not found in the initial tumor biopsy showed elevated levels after paclitaxel chemotherapy despite decrease of a PIK3CA mutation AF during stable disease. This suggests clonally divergent treatment responses. ctDNA testing achieved higher sensitivity and better correlation with tumor burden than CA15-3 or CTC count (74). Major limitations were the small sample size and that somatic mutations and structural genetic variants could be identified in only 60% of primaries. The limited clinical utility of CA15-3 was repeatedly reported (4, 163, 164). Chromosomal rearrangements were retrospectively investigated by WGS in resection specimens of 20 patients with surgically treated, otherwise therapy-naive primary BC (stages I–III) (75). These rearrangements were targeted by ddPCR assays for 4–6 patient-specific changes. Plasma samples were taken at surgery and three to six times during follow-up. Detection of ctDNA in ≥1 samples indicated relapse. Again, ctDNA positivity preceded clinical detection of metastatic growth in 86% (12/14) of patients with an average lead time of 11 months, while ctDNA remained undetectable after surgery in long-term disease-free patients (93% sensitivity, 100% specificity), making ctDNA a significant predictor of short RFS (75). Somatic alterations of archival primary tumor tissue and synchronous liver metastases collected at the time of diagnosis, as well as serial plasma samples collected during fourth line treatment with an AKT inhibitor (ipatasertib) were analyzed in a single patient with estrogen receptor (ER) positive, HER2-negative invasive mixed ductal–lobular adenocarcinoma of the breast. Sequencing was targeted on the high-depth massively parallel sequencing platform Integrated Mutation Profiling of Actionable Cancer Targets that comprises 300 cancer genes with actionable mutations (7). Parallel ctDNA analysis revealed mutations from both, primary and metastatic sites. Changes in ctDNA mutant AF mirrored the pharmacodynamic response to targeted monotherapy (7). This case study points out that ctDNA can deliver a holistic view of the tumoral genetic landscape. Genetic aberrations occurring under sequential targeted treatment with tamoxifen and trastuzumab, followed by lapatinib in a metastatic ER-positive, HER2-positive BC patient were tracked during 3-years. Stem mutations that were present in all tumor biopsies had highest allelic plasma levels, followed by metastatic-clade and private mutations. Serial changes in ctDNA subclonal private mutations correlated with individual treatment responses of metastatic sites (5). Similarly, primary tumor WGS identified somatic mutations in mBC patients undergoing two phases of chemotherapy (epirubicin and paclitaxel). The dynamics of 10 selected mutations from the primary were followed in serial plasma samples before and after treatment. A sharp decline in AF under therapy and an increase at disease progression were reported (165). Other studies confirmed that ctDNA contains mutations from both primary and metastases in BC (7, 166). The mutation profile of primary tumor, liver metastatic site, and plasma ctDNA of a metastatic ER+/HER2+ BC patient was studied by WES (166): The primary tumor was biopsied 4 months after neoadjuvant chemotherapy. Plasma and liver metastasis were sampled after progression following a two-phase therapy (anastrozole and herceptin). AFs of mutations in cfDNA correlated well with liver metastasis but poorly with the primary tumor. Moreover, the resistance mutation ESR1(p.D538G) in response to estrogen deprivation was detected in cfDNA and metastatic site only, but not in the primary, indicating a subclonal change under selective pressure by the aromatase inhibitor anastrozole. Likewise, the PIK3CA(p.H1047R) mutation was present in the primary only, suggesting that it emerged either after metastatic spread or that it was not present in subpopulation of cells responsible for metastatic seeding (166). Activating mutations in the estrogen receptor 1 (ESR1) gene are acquired under treatment in approximately 20% of patients and drive resistance to antihormonal therapy. Such mutations are predictive of endocrine resistance in mBC. Serial plasma ctDNA samples from 48 ER+ mBC patients receiving antiestrogenic therapy were analyzed by targeted NGS and ddPCR. ESR1 mutations were present in 3/48 patients at baseline with variable AF (p.D538G: 46.3%, p.Y537S: 2.8%, p.E380Q: 24.4%). In four patients, an ESR1 resistance mutation was detected in cfDNA under therapy (76). Comparable data were reported by others (77). However, the abovementioned case studies comprise small patient numbers and the identification of breast cancer driver mutations remains challenging with only a few being identified (HER2, TP53, PIK3CA, and AKT1; amplification of ERBB2 and EGFR). Thus, WGS or WES might aid in identification of unknown oncogenic drivers. To date, such a strategy would lead to a significant cost increase in diagnostics and requires extensive bioinformatics support typically unavailable in routine labs. Despite availability of WGS/WES, discrimination between biologically relevant somatic driver mutations, passengers, and background alterations remains difficult. Large prospective clinical trials are required to evaluate if and to what extent early detection of metastasis by ctDNA monitoring might improve patient outcome. Nevertheless, case study data promote ctDNA testing to facilitate early therapeutic intervention while tumor burden is still low.

#### Lung Cancer

While surgery offers best curative possibilities in early-stage NSCLC, it is usually not an option in advanced disease. Besides cytostatics, targeted therapies significantly improved clinical outcome in LAC. A number of molecular targets was identified so far in these tumors (EGFR, ALK, HER2, BRAF, MET, ROS1, and RET) but only few in LSCC (FGFR1 and PIK3CA). Targeted therapy choice depends on the molecular profile of the primary. However, despite initial response, almost all tumors become resistant within short time. To date, molecular testing is routinely performed on tumor tissue, typically needle biopsies. 41–62% of LC patients receiving 1st and 2nd generation EGFR TKIs acquire resistance after ~12 months due to the EGFR(p.T790M) mutation (167). Osimertinib is a 3rd generation TKI used to treat metastatic NSCLC patients carrying EGFR(p.T790M). Unfortunately, EGFR-mutated tumors can escape EGFR blockade in several other ways: by amplification of the MET receptor tyrosine kinase or ERBB2, by mutations in PIK3CA and BRAF, as well as by activation of AXL and NFkB (167). KRAS mutations in the tumor are a negative predictor of response to EGFR-TKIs or anti-EGFR antibodies (168). Therefore, continuous monitoring of treatment effects and arising resistance during follow-up is of high clinical relevance. Recently, the first ctDNA screening test (cobas^®^ EGFR Mutation Test v2, Roche, Switzerland) for molecular analysis of ctDNA in metastatic NSCLC patients in whom mutation screening was impossible in tumor tissue was approved by the FDA. This test detects a series of activating EGFR mutations–exon 19 deletions and p.L858R/p.T790M point mutations—in cfDNA, identifying patients who might benefit from TKI therapy in oncological diagnostic routine. cfDNA was analyzed for the presence of p.T790M mutation and patient-specific EGFR activating mutations known from primaries by BEAMing in a cohort of 23 advanced NSCLC cases progressive after EGFR-TKI treatment and 21 advanced NSCLC, EGFR-TKIs-naive patients (99). Most patients had already progressed to stage IV. The p.T790M mutation was detected in 43.5% of EGFR-TKI treated patients at an AF from 0.1 to 1%. Similar findings were reported independently (49, 100). In the phase I AURA study assessing safety, tolerability, and efficacy of osimertinib in EGFR mutant NSCLC progressive under EGFR-TKIs, plasma p.T790M genotyping in 237 patients revealed 70% sensitivity and 69% specificity. ctDNA p.T790M-positive patients had similar outcomes as those with the same mutation detected in tumor tissue. By contrast, p.T790M plasma negativity and tumor tissue positivity correlated with a favorable outcome which, in conclusion, should prompt for a confirmatory tumor biopsy to avoid false-negatives in case p.T790M-negative plasma (101). Correlation between OS and dynamic changes in AFs of EGFR(p.T790M) before and after EGFR-TKI therapy was addressed in a prospective cohort of 103 advanced-stage NSCLC patients (102). Plasma samples before and after EGFR-TKI therapy were analyzed by dPCR array chip (Fluidigm, South San Francisco, CA, USA) and ARMS. Patients with the EGFR(p.T790M) in pre-TKI plasma samples had inferior PFS and OS compared to those without the mutation. Patients with an EGFR-sensitizing mutation and high AF of pre-TKI p.T790M had a shorter PFS (*p* = 0.001) under EGFR-TKI compared to those with a low AF (102). The resistance mutation EGFR(*p*.C797S) was identified in cfDNA by NGS of 15 EGFR-TKI ADZ9291-treated patients whose tumors were positive for EGFR(p.T790M) (169). Besides EGFR(p.T790M), a range of driver and resistance mutations/aberrations, including ALK, ROS1, and RET rearrangements, HER2 insertions, and MET amplification have been identified in pretreatment plasma of progressive NSCLC patients by NGS with 100% specificity and 77% sensitivity (170). Similarly, in a study on 102 prospectively enrolled NSCLC patients (81% LAC, 96% stage IV) driver and resistance mutations were identified by ctDNA NGS (92): Concordance between tumor tissue DNA and ctDNA correlates is higher with shorter intervals between tissue and blood sampling (*p* = 0.038) (92). In sum, plasma ctDNA testing in LC is beneficial for therapy selection and more feasible than serial tissue biopsies.

The aim of the large prospective TRACER× trial (TRAcking non-small lung Cancer Evolution through therapy R[x]) on 842 NSCLC patients (stages I–IIIA) is to monitor clonal evolution from initial diagnosis to death by analysis of multiple genomic regions in tumor tissue, cfDNA, and CTCs (171, 172). Analysis of the first 100 patients detected ctDNA in 48% (46/96) during early stages. Predictors of ctDNA-positivity were non-LAC histology, increased proliferative indices, and lymphovascular invasion (103). Clonal mutations in tumor tissue were identified in 100% and subclonal ones in 68% of ctDNA-positive patients. Tumor volume and clonal variant mean AF correlated directly (103). Hence, prior knowledge of clonal variants for ctDNA screening is more sensitive than tracking subclonal variants. Pre- and postsurgical ctDNA profiling with patient-specific gene panels during follow-up confirmed SNVs in 93% (13/14) of patients with morphological or clinical relapse. The median interval between ctDNA occurrence and later CT-morphological relapse was 70 days (103). High amounts of different mutations in ctDNA of LC patients is associated with poor OS (173).

#### Colorectal Cancer

Significant proportions of CRCs harbor mutations in KRAS, BRAF, or NRAS that are negative predictors for an EGFR-blockade therapy, making these hotspots an appealing diagnostic cfDNA target (117, 138). Direct correlation between KRAS ctDNA AF and OS was found (4). Metastatic lesion-specific radiographic responses to targeted therapies in CRC can be driven by distinct resistance mechanisms affecting MAPK pathway genes which can arise asynchronously in separate lesions in a single patient (174). Following initial therapy for stage IIIa CRC, a patient experienced relapse and new liver metastases. Upon progression, the patient received an EGFR blockade with cetuximab and panitumumab. While the TP53(p.E171*) mutation was identified in the primary, the resistance mutation MAP2K1(p.K57T) upon EGFR-blockade arose in only one liver metastasis. Response to EGFR-blockade was objectified by a decrease in size of the primary and one liver metastasis. Shortly after, the neighboring liver metastasis increased and ctDNA analysis now identified KRAS(p.Q61H) which was not present in either primary tumor now or in the responding liver metastasis, suggesting that it was present even before EGFR-blockade (174), illustrating that single tumor biopsies may not sufficiently represent tumor heterogeneity. Overall outcome depends on lesion-specific therapy responses. Patients with RAS wild-type CRC primaries and ctDNA positive for KRAS and BRAF mutations were resistant to the EGFR-blockade (4, 121, 122, 163). Such mutations can be detected in the blood of cetuximab or panitumumab treated patients as early as 10 months before clinical disease progression (121, 163). Interestingly, KRAS resistance clones, which emerge in blood during EGFR blockade, can even decline upon withdrawal of anti-EGFR antibodies, allowing for rechallenge that can again lead to response (122).

A multicenter prospective study on 53 metastatic chemotherapy-naive CRC patients analyzed ctDNA level as early therapy response marker. Plasma before, 3 days after surgery, and before the 2nd chemotherapy cycle revealed no significant difference before and 3 days after surgery. Reduction from presurgical to pre-chemotherapy level predicted radiographic response better than absolute levels with no significant correlation between ctDNA fold change and OS (123). ctDNA for MRD detection was prospectively tracked in 18 resected CRC patients. Plasma was serially sampled over 12 weeks postoperatively. Tumor-specific mutations were identified from archival FFPE tissue and individually selected mutations were tracked by BEAMing in plasma. No recurrence was observed if ctDNA was undetectable 2 weeks after surgery. By contrast, ctDNA positivity in postoperative plasma was predictive of relapse. A sharp drop in ctDNA 2 to 10 days after surgery was observed after complete as opposed to incomplete resection (48).

## Circulating Tumor Cells

### Biological Significance and Origin of CTCs

Circulating tumor cells are cancer cells, detached from tumor tissue, floating in the bloodstream, bearing the potential to seed the disease to other sites, as demonstrated half a century ago (175). Metastatic progression comprises of four steps: local invasion, intravasation, extravasation, and colonization (176, 177). CTC precursors can remodel the surrounding stroma by activation of extracellular proteases allowing them to overcome the basement membrane and extracellular matrix to which they normally adhere (178). A subpopulation of precursor carcinomatous CTCs can undergo partial or complete EMT, e.g., by repressing expression of E-cadherin and cytokeratin as well as inducing vimentin and N-cadherin. Thereby, they detach from epithelial sheets to become invasive and motile. Once they invade blood vessels within the tumor microenvironment they can become CTCs (179). Most CTCs will not survive because of anoikis (apoptosis due to vanished cell–matrix interactions), shearing forces of blood flow, and immune cell attack (180). Once lodged in blood vessels of distant organs, CTCs can extravasate and infiltrate the surroundings (178). During colonization, CTCs resume growth in a distant organ to form a metastasis, undergo cell death or enter dormancy (181). CTC have been isolated from blood as either single cells, or clusters. In breast and prostate cancer, CTC clusters were shown to consist of oligoclonal cells from the primary and are associated with higher metastatic potential than single CTCs (182). CTC clusters contain either just a group of neoplastic cells or are associated with fibroblasts, leukocytes, endothelial cells, and platelets (183). In the next section, we will review the technologies for CTC detection and enrichment, followed by a critical appraisal of the significance of CTC enumeration in breast, lung, and colorectal cancer.

### CTC Detection and Enrichment Technologies

The key challenges in CTC isolation are their rarity in blood (1–10 CTCs per 10 ml) and lack of cancer-specific surface markers. Hence, a multitude of enrichment technologies have been developed. CTCs can be positively or negatively enriched based on their biological or physical properties. Methods based on biological properties use antibodies that bind surface markers on CTCs. For detection of epithelial CTCs, antibodies against EpCAM and cytokeratins (CK8, CK18, CK19) are frequently used, while mesenchymal CTCs can be selected by antibodies against N-cadherin and vimentin. Cell Search is so far the only platform approved by the FDA for clinical use. Epithelial CTC enrichment is based on positive selection *via* EpCAM antibody-coated ferromagnetic beads with subsequent staining with DAPI, anti-CD45, and anti-cytokeratin to identify and enumerate CTCs. CTCs can be negatively enriched by antibodies against CD45 to deplete leukocytes from a blood sample. Alternative enrichment strategies are based on physical properties, namely size, deformability, density, and electric charge. Detailed descriptions of these technologies have already been summarized (184). Epithelial antigen-based positive or negative selection is limited due to their inability to detect carcinoma CTCs after EMT. Hence, these approaches can produce false-negative results as opposed to biophysical methods. However, in the latter, blood cells can have properties similar to CTCs, resulting in high false-positive rates. To date, no single method is able to capture the entire spectrum of CTCs. Due to numerous different isolation approaches and lack of multicenter validation, their robustness, reproducibility, sensitivity, and specificity remain elusive. Currently, the method of choice depends on tumor type and intended downstream analyses: genetic studies require high purity, FISH and immunofluorescence high capture efficiency, and drug testing even viable CTCs. Hence, similar to cfDNA, development of standardized protocols for sample handling, sample storage, enrichment, enumeration, and evaluation are important.

### CTC in Cancer Patient Management

The prognostic value of CTC enumeration was demonstrated in BC, LC, CRC, and mCRPC (177). While in patients with BC (78, 185), mCRPC (186), and NSCLC (104) a cut-off value of ≥5 CTC per 7.5 ml of blood indicates worse prognosis, in CRC a cut-off of ≥3 CTC per 7.5 ml of blood is predictive of shorter OS (124). Higher CTC counts in pulmonary vein and mesenteric blood than in peripheral blood of LC and CRC patients, respectively, have been reported (187, 188). In the next section, we will discuss prognostic value of CTC number and use of CTC in monitoring the effect of anticancer therapy.

#### Breast Cancer

A prospective study on early-stage BC investigated the prognostic value of CTC number for OS in 2,026 patients before and 1,492 after adjuvant chemotherapy using CellSearch. The patients were followed over a median of 35 months. CTCs before chemotherapy were detected in 21.5% and post-therapeutically in 22.5% of patients. Their presence before and after chemotherapy was associated with shorter RFS (*p* < 0.0001), BC-specific survival (*p* < 0.008) and OS (*p* < 0.0002). In metastatic BC, ≥5 CTCs per 7.5 ml of blood before therapy start and at first follow-up was predictive of shorter PFS/OS and correlated with lymph node metastasis (78). The predictive value was independent of time to and site of metastasis, as well as hormone receptor status (79). Other studies confirmed the prognostic value of CTC counts concerning PFS and OS in early-stage and metastatic BC (189, 190). The SWOG S050 randomized clinical trial investigated the value of CTC enumeration in monitoring chemotherapy response in mBC, in particular, if an early switch in first-line regimen would improve OS in those subjects with increasing CTCs under the primary drug (80). Randomization was between continuation of the initial treatment and therapy change. No difference in median OS was observed in the high-risk group despite the change of chemotherapy. Based on this finding, the American Oncology Society clinical practice guidelines for CTCs considered not to use CTC count in mBC management (191). However, median OS for low, moderate, and high-risk groups were 35, 23, and 13 months, respectively. This study confirmed the prognostic but not predictive value of CTC counts in mBC patients receiving first-line chemotherapy (80). The prognostic value of CTCs in 44 HER2-positive mBC patients with cerebral metastases not previously treated with whole brain radiotherapy under HER2-targeted treatment (lapatinib, capecitabine) was investigated as a part of the LANDSCAPE clinical trial. CTC number was analyzed at baseline and at day 21 before the second therapy cycle. Objective CNS response was significantly higher in patients who did not have any CTC detected at day 21 (85). However, others showed the inability of CTCs to predict the risk for cancer dissemination and that the prognostic value of CTC detection depends on the test method (81, 192). Single CTCs and pooled CTCs were analyzed for PIK3CA mutations in 18 mBC patients. Analysis of single CTCs in two patients revealed different PIK3CA mutations in single CTCs (82). If CTCs would reflect biologically relevant clones with regard to treatment, then the heterogeneity between single CTCs in an individual patient limits the usefulness of their genetic analysis in diagnostic settings. CTCs have been frequently detected in HER2+ primary tumors of mBC patients (193). HER2+ CTCs have high metastatic potential (83) and were found in 89% of patients with HER2-negative primaries (84). Residual CTC clusters at days 15 and 29 of therapy (nab-paclitaxel with or without tigatuzumab), but not at baseline, predicted shorter PFS (194).

#### Lung Cancer

Prognostically relevant cut-off value for CTC counts by CellSearch in NSCLC was defined as ≥5 CTCs/7.5 ml blood, and in SCLC as ≥50 CTCs/7.5 ml (104, 195). In a study on 208 NSCLC patients (stages I–IV), 50% of cases had CTCs detected preoperatively by ISET filtration enrichment. Here, a cut-off of ≥50 CTCs/7.5 ml blood significantly correlated with decreased DFS and OS in early and advanced-stage NSCLC. CTC counts did not correlate with tumor stage, age, gender, tobacco exposure, tumor size, and malignant pleural effusion (105). Serial CTC enumeration showed correlation of a low count with radiographic tumor response, while increased numbers reflected tumor progression. Therefore, CTC number might be used as pharmacodynamic marker where change in CTC count during therapy would inform about response (106).

The prognostic value of CTCs in early NSCLC was assessed in a cohort of 168 chronic obstructive pulmonary disease patients. Five CTC-positive patients (5/168) developed lung nodules 1–4 years after CTC detection, 4/5 were diagnosed with LAC, and 1/5 with LSCC. However, three more formally CTC-positive subjects did not develop any neoplasm during follow-up (107), highlighting a relatively high false-positive rate that limits clinical applicability.

High concordance rates in EGFR mutations in NSCLC patients (84%, 31/37) between primaries and CTCs were observed (108). 92% concordance was found in another study (106). A classifier based on CNVs in single and pooled CTCs from 31 pretreatment SCLCs distinguished chemosensitive from chemorefractory cases (cisplatin and etoposide) with 83.3% accuracy. This study concluded that chemoresistance occurring under therapy differs from *de novo* resistance since five patients that initially responded and then relapsed had the same CNV profiles before and after relapse (109). CTCs were present in 85% of 97 LSCC patients (range 0–44,896/7.5 ml blood) at baseline before chemotherapy. CTC clusters and CTCs with apoptotic morphology were detected in 32 and 57% of patients, respectively. Baseline CTC count and change in number after one cycle of chemotherapy were independent prognostic factors in SCLC. The numbers of CTCs and CTC clusters correlated with stage, serum LDH, presence of liver metastases, and number of metastatic sites (195).

#### Colorectal Cancer

A prospective, multicenter study on 430 mCRC patients evaluated the prognostic and predictive value of CTC counts by CellSearch at baseline and after three lines of therapy. ≥ 3 CTCs/7.5 ml blood at baseline or during follow-up were an independent prognostic factor of poor PFS and OS (124). Screening for EMT in CTCs in 1,203 CRC patients by CanPatrol™ enrichment with analysis of epithelial (EpCAM, cytokeratins), and mesenchymal (VIM, TWIST, AKT2, SNAI1) markers revealed three distinct phenotypes: epithelial, mesenchymal, and biphenotypic. CTCs were detectable in 86.9% of patients. Total CTC counts correlated with clinical stage, lymph node, and distant metastases. Biphenotypic and mesenchymal phenotype counts correlated with tumor stage, suggesting that CTCs with EMT have a higher metastatic potential and are more aggressive (125). Preoperative CTC detection (≥1 CTC/7.5ml blood) is an independent prognostic factor for disease progression and OS in patients with non-metastatic CRC (126).

KRAS analysis in primaries is mandatory before starting EGFR-targeted therapy in mCRC. Concordance rate in KRAS state between CTCs and primaries was 77% (128) while only 50% were found in another study (196). This discordance between CTCs and primaries might be due to intratumoral heterogeneity and multiple metastatic clones that disseminate during early disease stages that might remain dormant for years (127).

The usefulness of KRAS testing in CTCs before curative surgery was addressed in 35 mCRC cases at various tumor stages. CTCs were captured based on their size and analyzed for KRAS mutations by ddPCR. 90% of patients harbored at least one CTC/3ml blood; in 7% of cases, CTC clusters were detected. CTC counts correlated with disease stage, but not with serum concentrations of tumor markers, CEA and CA19.9. CTCs of 57% patients were positive for one of the relevant KRAS mutations in codon 12 or 13 (128). Heterogeneity in EGFR expression, mutations in PIK3CA and KRAS between CTCs of the same mCRC patient was observed (127).

## Comments and Future Perspectives

cfDNA quantification appears unsuited for cancer-specific questions, as both sensitivity and specificity are low. Numerous studies suggest a clinical usefulness of ctDNA testing as a prognostic, predictive and diagnostic biomarker in various neoplasms. Concordance, though not 100%, in frequencies of mutations found in tumor tissue and cfDNA, with significantly lower allele frequencies in cfDNA, have been described in many studies. Therefore, there is a need for development of highly sensitive analytic methods able to detect genetic alterations at low allelic frequencies in plasma. However, this implies a certain unspecific detection rate, partially impairing diagnostic routine use. As shown in several studies, combination of single or several tumor-associated genetic alterations found in cfDNA with other biomarkers and imaging can increase diagnostic specificity and sensitivity. In clinics, ctDNA analysis will most likely be used complementarily to imaging for monitoring disease burden as it can not deliver precise information about tumor location. Thus, we foresee that a combination of genetic analysis of tumor tissue and ctDNA together with medical imaging may deliver increased accuracy in determination of metastatic growth. Ultimately, tumor surveillance *via* blood samples, periodically drawn by physicians also in remote locations and sent to the treating center, could be of significant benefit for the patients. Importantly, analytic and clinical validity of ctDNA remains to be demonstrated. The mechanisms of cfDNA release into the bloodstream still remain elusive. Whether all metastatic sites contribute similarly to the ctDNA pool remains unknown. Overall tumor burden appears to be better represented when restricting ctDNA tests to previously known tumor-specific mutant alleles, typically those derived from molecular workup of tumor biopsies with emphasis on driver mutations that are less likely to change during tumor evolution. Qualitative cfDNA analysis may inform of tumor heterogeneity and detect emerging secondary resistance mutations. Detection and quantification of EGFR(p.T790M) mutation in ctDNA might be used as a predictive biomarker under EGFR-TKI treatment of NSCLC patients that could influence treatment changes. However, such clinical evidence has not been demonstrated for other mutations. As with genetic data from tumor tissue, it remains debatable how to deal with aberrations for which no targeted therapies are available. Unfortunately, knowledge about likely progressive disease before deterioration of clinical symptoms may not even be beneficial for quality of life and would not necessarily prolong OS. Most importantly, there is an urgent need to elucidate origin, function, and biological significance of cfDNA before implementing it in routine diagnostics.

The lack of detection at diagnosis or at time of progression in some patients is a key limitation to the use of ctDNA as prognostic marker. Therefore, at this time, we believe that analysis of ctDNA genetic alterations will and should not replace tumor biopsy or radiological evaluation. It can, however, significantly improve clinical follow-up by monitoring treatment efficacy more specifically than imaging. While ctDNA levels might correlate with tumor volume, total cfDNA reflects tumor burden, mirroring overall disease biology. We envision that clinical laboratories will need to provide deep sequencing analysis to define clonal mutation prevalence, focusing on actionable genes and mutations as well as those considered hallmarks of cancer, e.g., KRAS mutations to stratify patients with CRC for anti-EGFR therapy, driver mutations to be tested in plasma for tumor monitoring, and therapy response as well as EGFR mutations to predict benefit from erlotinib, gefitinib, and osimertinib.

Despite technological advance in CTC isolation and analytic methods, the biological fate and significance of these cells remains elusive. It may be expected that they represent a highly heterogeneous population. In fact, antibody-based detection of single tumor cells—not in blood but lymph nodes—has led to substantial effort in routine diagnostics due to the introduction of respective TNM category pN0[i+] (197). However, this category was recently removed (198). Lymph nodes with immunohistochemically detected isolated tumor cells are now only tabulated in the report but do no longer contribute to overall N classification (199). While ctDNA represents a potpourri of many, if not all tumor sites in a patient, CTC-derived nucleic acids are much lower in copies and, hence, may not improve diagnostics over conventional biopsies while ctDNA tests are very likely to proliferate in clinical practice. As for cfDNA, there are several drawbacks in CTC research that limit usefulness of CTC counting in clinics: (i) different methods have been employed for CTC enumeration limiting the comparison of the data across studies. (ii) Reproducibility and sensitivity of these methods has not been thoroughly determined. (iii) All currently available methods are tumor type-specific, i.e., mostly epithelial cancer cells, but not those after EMT are selected while the latter may be of high biological relevance (4). It is still impossible to determine aggressiveness of single CTCs and CTC clusters (5). No universal signatures of CTCs have been identified—should they even exist—that would cover any stage and type of cancer.

In conclusion, we believe that liquid biopsies are likely to become an additional standard for monitoring progressive genomic alterations over tumor evolution during exposure to targeted therapies. They also might prove effective in cases where obtaining tumor tissue would have a high risk of clinical deterioration. For the majority of cases, however, we envision liquid biopsy as second-line diagnostic tool, building on the findings of morphological, genetic, and epigenetic changes derived from classical tissue biopsies, eliminating their shortcoming in holistic, as well as spatio-temporal understanding of each neoplasm in a personalized, patient-oriented manner.

## Nomenclature

**Table:** 

AC	adenocarcinoma
AF	allelic frequency
cfDNA	cell-free DNA
ctDNA	circulating tumor DNA
CNS	central nervous system
CNV	copy number variation
CRC	colorectal cancer
CTC	circulating tumor cell
BAC	bronchoalveolar carcinoma
BC	breast cancer
BCSS	breast cancer-specific survival
DFS	disease-free survival
DNA	desoxyribonucleic acid
eBC	early-stage breast cancer
EMT	epithelial–mesenchymal transition
FDA	U.S. Food and Drug Administration
dPCR	digital polymerase chain reaction
ddPCR	droplet digital PCR
iDES	integrated digital error suppression
HCC	hepatocellular carcinoma patients
HGIN	high-grade intraepithelial lesions
LAC	lung adenocarcinoma
LC	lung cancer
LCC	large cell carcinoma
LDH	lactate dehydrogenase
LOH	loss of heterozygosity
LSCC	lung squamous cell cancer
mBC	metastatic breast cancer
mCRC	metastatic colorectal cancer
mCRPC	metastatic castration-resistant prostate cancer
MRD	minimal residual disease
NGS	next-generation sequencing, massively parallel sequencing
NSCLC	non-small cell lung cancer
NSE	neuron-specific enolase
OS	overall survival
PCR	polymerase chain reaction
PFS	progression-free survival
PSA	prostate-specific antigen
PPV	positive predictive value
qPCR	quantitative polymerase chain reaction, also termed real-time PCR
RFS	recurrence-free survival
SCLC	small cell lung cancer
SNV	single-nucleotide variant
tDNA	tumor DNA (isolated from tumor tissue directly)
TKI	tyrosine kinase inhibitor
TNBC	triple-negative (estrogen and progesterone receptor, and Her-2)
UID	unique identifiers
WES	whole-genome sequencing
WGS	whole-exome sequencing

## Author Contributions

IH, JH, and MT have contributed equally to write this manuscript.

## Conflict of Interest Statement

The authors declare that the article was written in the absence of any commercial or financial relationships that could be construed as a potential conflict of interest.
